# Therapeutics of Angina

**Published:** 1885-11

**Authors:** A. Gerald Hall


### THERAPEUTICS OF ANGINA.—SORE THROAT.

The treatment of the different forms of angina is generally directed with reference to their characteristics, their intensity, and complications. The first duty of the physician in these, as in all acute diseases, is to arrest their probable causes, and to remove every agency capable of augmenting these maladies; then to prescribe a suitable diet, which most frequently consists in abstinence from all aliment; and finally to select the remedy most apposite to the case.

*Actæa* is serviceable whenever there occurs stiffness of the neck, a sensation of swelling and vehement pressure in the tonsils; great dryness and burning heat in the throat, with a sensation of hot air passing over it; extreme sensibility of the throat to cold drinks and to cold air; burning itching; contraction in the throat on swallowing solid food; painful pressing after having spoken; irritation followed by cough and bloody expectoration. When these symptoms persist, despite the previous use of Aconite, the Actæa, aided by Nux vomica, will mitigate them in a few days.

*Ammonium carbonicum* applies as a remedy when there is burning on the neck, extending as far as the throat; sensation of swelling in the tonsils on swallowing; pressure with congestion of the œsophagus, as if some substance had been arrested in its passage, although exempt from pain; speaking difficult; voice hoarse; nocturnal cough violent; respiration short; and occasionally an aphthoüs appearance. Great sensibility against cold, weakness of the limbs, a continued shudder, which alternates, at night, with heat, also indicate this remedy.

*Ammonium muriaticum* is indicated by shooting pains in the neck, whether on swallowing, or independent of deglutition, and also in the throat on gaping, with bitter taste, anorexia, unquenchable thirst, dry cough, and dry coryza; when frequent tickling is joined to a sensation of roughness and shooting pain, and to a great dryness of the throat, at the same time that there arises an abundant secretion of mucus, which it is very difficult to expectorate. Then occur repeated shudders, great fatigue, and flushes of agonizing heat. The Sal ammoniac also answers when the malady is obstinate and threatens to pass over to a chronic state.

*Baryta* benefits when there are penetrating pains in the throat on empty swallowing; pressure and shooting pains on swallowing aliments; strong swelling suppuration of the palate and tonsils; obstructions to speech and deglutition; sometimes, in the morning, dryness and painful stitches on swallowing, recurring at night; contraction of the throat, with labored respiration after meals; efforts to belch; scratching in the throat; humid coryza, with dry cough, alternate chills, and flushes of heat. The Baryta renders the greatest service when the angina lingers, remains stationary, passes over to the chronic state, or resembles schirrus.

*Belladonna* is to be given if a violent fever and burning heat accompanies the pains of the throat, with warmth and swelling of the veins; if there is dryness of the throat and mouth; shooting pains in the throat on swallowing, turning the head, or breathing; or when on swallowing there is experienced a sensation of a bruise and burn, or, in addition, a contraction and oppression of the throat, which impedes the deglutition, speech, and respiration. *Dry* cough is an important indication of Belladonna, and, also, swelling of the tonsils. It frequently succeeds Aconite with marked benefit.

*Bryonia* accords with pricking sensations in the throat on swallowing and turning the head; pressure, swelling and dryness of the back of the throat, the palate, and mouth; abundant secretion of saliva, constipation; cold in the head and hoarseness; dry cough and oppressed respiration. Bryonia follows the Aconite advantageously in practice.

*Cainca* has been applied with the greatest success when the salivation has been abundant; when there has been swelling of the uvula and palate with a grating sensation; a constant contraction in the throat alternating with drawing; heat; difficulty of deglutition; hollow, oppressed, and hoarse voice; pressure on the larynx; copious expectoration of watery mucus; sneezing; dry cough; difficulty of respiration at night; swelling and paleness of the face. This remedy is remarkably active and curative in many catarrhal forms of angina, especially those which precede scarlatina and measles. It is also useful in the treatment of anasarca that succeeds these two diseases.

*Cantharides* deserves employment when the throat manifests a burning and grating sensation; when there is redness and tension in the mouth; or pressure terminating in shooting pains on swallowing; or when the patient cannot swallow liquids; has a bitter and sour taste; white tongue; salivation; violent tickling in the larynx; dry cough, sometimes followed by bloody expectoration, and labored, painful respiration. Cantharides has proved useful at the conclusion of inflammatory, and at the commencement of catarrhal sore throats.

*Capsicum* is an energetic remedy when an inflammatory pain exists in the throat, which becomes drawing, or very contracting and convulsive, irrespective of the deglutition; when a painful pressure, a kind of contraction, exists in the curtain of the palate during deglutition, and when the ganglions of the neck experience rending and agonizing pains, recurring by paroxysms. To these symptoms may be added tickling in the throat, which causes frequent sneezing and sensation of roughness; weak, disagreeable taste; excretion of abundant and thin mucus from the nose; hoarseness; dry, hacking cough, and the production of a copious mucus in the trachea, expelled by expectoration. *Capsicum* is also appropriate to many epidemic maladies, or to such of its indications as occur suddenly during the prevalence of an epidemic. Sore throats complicated with gastric or rheumatic ailments, as well as those of unfavorable forms that pass over suddenly to a gangrenous state, yield readily to *Capsicum*, given twice in six hours.

*Chamomilla* responds expressly to angina complicated with gastric and bilious fever; also with painful deglutition; a sensation of fixed pain in the throat; bitter taste; malaise; nausea, and catarrhal affections, particularly dry coryza, tickling in the larynx, hoarseness, dry cough, and difficult respiration. *Chamomilla* is indicated in all mucous diseases, and therefore is especially suitable for catarrhal sore throats.

*Cocculus* is applicable to dryness of the mouth, with a sensation of roughness in the throat, or burning in the throat which extends quite to the curtain of the palate, with a flow of saliva, very great sensibility of the neck, even to smarting, pressing pain in the tonsils on swallowing, bitter and offensive taste, distaste for all aliment, partial paralysis of the œsophagus, with sensation of inability to swallow, contraction of the throat, difficulty of respiration and irritation constantly inducing cough; at night the cough becomes violent and menaces suffocation. *Cocculus*, after the prior administration of *Aconite*, will relieve all inflammatory traces of the above symptoms that the latter remedy does not reach.

*Drosera* is an admirable remedy for dryness and contraction of the palate and pharynx; pricking in the throat, without deglutition; expectoration of watery saliva; irritation to cough, with darting and pricking pains in the larynx, hoarseness, yellow mucous expectoration, and difficult respiration. The voice becomes materially changed, and the cough, which occurs in the evening on retiring and during the night, is developed in deep, repeated, and convulsive paroxysms, which are sometimes succeeded by vomiting.

*Hepar.*—Pricking sensations in the throat as if from pins, on swallowing, gaping, respiring deeply, and turning the head, which sometimes extend to the ears; pain, as if from a bruise, in the muscles of the neck; interiorly a sensation of swelling and pressure as if from some fixed external body; sensation of scraping on swallowing solid aliment; heat and scraping in the throat after primary relief, with constant expectoration of mucus; vomiturition in the morning, with a dry and deep cough, which develops itself at evening and becomes sometimes extremely violent and agonizing; frequent expectoration of mucus and blood; expression of the face weakened; eyes black and blue, shivering followed by heat, clammy perspiration, especially on the forehead and chest—such are the symptoms which indicate the use of this remedy. In sore throats of the most serious character, which threaten to destroy by suffocation, also in croup, the Hepar sulphuris displays the most astonishing power, and not less conspicuously where there is a tendency to induration of the tonsils, or when such state is developed. The *third* potence of this medicine has acquired a decided preference, for efficacy, over any other of its forms.

*Hyoscyamus* is in requisition for burning heat in the face, the features of which are distorted, and the complexion purplish; for dryness of the throat, thirst, prickings in the larynx, contraction of the throat, impossibility of swallowing, copious salivation, increasing loss of appetite; for vomitings of white mucus or of green bile, collection of mucus in the larynx and trachea, hoarse and indistinct voice connected with a sensation of a foreign body firmly lodged in the trachea, nocturnal cough, which may be dry and spasmodic, and respiration labored and agonizing. The Hyoscyamus is peculiarly suitable to sensitive and irritable constitutions disposed to spasms or convulsions.

*Ignatia.*—Lancinating pains in the throat, sensation of a foreign body lodged there, a bruised pain on swallowing, pricking in the windpipe, constant effort to swallow, pains in the cervical ganglions, pressure on the entire œsophagus, rending pains in the larynx which increase on swallowing, respiring, and coughing; sensation of ulceration of the nose with coryza, dryness of one nostril, copious secretion of mucus in the trachea, progressive tickling in the larynx with cough, or, in its place, difficult yellow expectoration with contraction of the throat that excites a cough; distention of the abdomen and constipation. The Ignatia not only accords with the previous state, but is of infinite importance in rheumatic and gastric constitutions. It frequently requires to be followed by *Pulsatilla, Rhus*, or some appropriate antipsoric remedy.

*Ipecacuanha.*—Rough, bruised, pricking, and swollen sensation of the throat, especially during deglutition ; elongation and painful sensibility of the palate ; liquid stools ; severe catarrh with drawing pains in the limbs ; violent cough with dyspnœa, and without expectoration, similar to whooping cough, with congestion of blood to the head, constriction of the surface joined to extreme paleness. *Ipecac.* is also useful in catarrhal sore throats, when they are connected with spasms of the chest and other nervous affections of the same nature. This medicine should be given every two days in alternation with *Nux vomica*, to which should be added *Arsenic* when agitation and dyspnœa supervene.

*Manganum aceticum* finds a place for the following symptoms : dryness, roughness, and a sensation of obstruction in the trachea; pain in the palate, without swallowing, with prickings on both sides of the neck on empty swallowing ; roughness of the throat, bitter, disagreeable taste, anorexia, hoarseness on inspiring air freely ; dry coryza, a disposition to cough, which modifies no other symptom ; dry cough after talking ; great dryness, roughness, and sensation of constriction in the larynx ; yellowish green mucous expectoration ; smarting extending to the cheeks ; febrile paroxysm at night. The *Acetate of manganese* has been amply tested in practice.

*Mercurius solubilis.*—Sensation as if from an obstruction in the throat, painful deglutition with pressure, constant effort to swallow, lancinating pains in the neck and in the tonsils extending to the ears, pressure in the œsophagus and larynx, which increases on eating ; drinks cannot pass by the epiglottis, and are forced back by the nose, swelling of the parotids and the cervical ganglions with pressing, burning, or lancinating pain, flow of thin and fœtid saliva, coryza and sneezing, dry and violent cough, occasional bloody expectorations, and dyspnœa. This remedy applies especially to scrofulous temperaments where the sore throats have endured for a long time without assuming a decisive character, or where there has arisen suppuration of the tonsils, palate, and pharynx ; or, further, an induration of the same. The Mercury also answers for the last stage of violent catarrhal sore throats which have been neglected or badly treated, and where the Eustachian tube is involved in these conditions.

*Nux vomica.*—Roughness and sensation of a bruise in the throat during deglutition and inspiration of cold air ; swelling of the curtain of the palate and of the uvula; pressing pains during and without swallowing; at the same time lancinating pains in the throat, which extend to the ears and submaxillary glands; sensation as if the smallest body would be arrested in the throat, on swallowing; burning heat in the larynx; grating sensation in the larynx; ulcerative pain in the nose, with an abundant secretion of mucus; itching and tickling in the larynx, cough on respiring and talking, rending in the jaw-bones and swelling of the face. This remedy is required in angina arising from colds, in which case *Aconite* should precede its use, if the inflammatory fever is very high. It also is remedial for simple catarrhal sore throats. The *Nux* is applicable when the swelling of the parts involved aggravates the disease to the danger of suffocation, especially when connected with obstinate constipation.

*Pulsatilla.*—Exterior lancinating pains on swallowing, pressure in the throat, sensation of swelling in the uvula and curtain of the palate; during and without swallowing, sensation of roughness and pain in the throat, or as if the swollen submaxillary glands throbbed with pulsating leaps forward, in the mouth; the palate seems filled with lumps, is painful on speaking and touching the tongue; in the morning an insupportable dryness of the throat, mouth, tongue, and lips; they become covered with a tenacious mucus, bad odor from the mouth and dry cough; in the morning, a flow of thick and yellow mucus, sometimes of fœtid matter, sensation of scraping and scratching in the throat, pain in the chest, perfect hoarseness, cough, with scratching and tickling in the trachea, copious and consistent expectoration, and dyspnœa.

*Stramonium* is indicated for extreme dryness of the throat, with inability to swallow, contraction, as if from a cord, altered voice, running into a very high octave, difficult speech, respiration exceedingly labored, anxiety, and blue discoloration of the face. This remedy should be also employed in spasmodic and convulsive conditions of angina, attended with exhaustion of the strength through the violence and duration of the malady.

*Senaga* responds to various indications; white tongue, mucous taste, vomiturition, smarting in the palate, inflammation of the pharynx and of the uvula, with enlargement; tension from the palate to the articulation of the jaws, dryness of the mouth and throat, collection of tenacious mucus, or of lumps of mucus, about the larynx; frequently a strong scratching, which compels the patient to expectorate and to swallow, with burning, itching, and pressing in the throat; also frequent sneezing, dry cough, or cough with expectoration of tenacious mucus, collection of mucus in the larynx, with tickling in the throat, dyspnœa, heat in the face, and slight chills. *Senega* is very useful in simple sore throats, as well as for rheumatic complications. The third potence is to be preferred.

*Sulphur* corresponds with strong heat in the throat from the larynx to the mouth, suppuration of the uvula and tonsils, or a sensation of elongation of the uvula, sensation of swelling and pressure in the throat, as if from a body lodged in the throat, especially on swallowing and respiring; sometimes lancinating pains and spasmodic constriction of the throat, as if the deglutition could not proceed below the gullet; also, dry coryza, hoarseness or total loss of voice, dyspnœa, and contraction of the chest. Although *Sulphur* may be specifically indicated for *chronic laryngeal phthisis*, it is also equally applicable to similar conditions of the throat; moreover, when symptoms slightly inflammatory have not been entirely removed by more active or acute remedies, when hoarseness persists, and when the general symptoms are unyielding and threaten a fatal termination.

We have only designated such remedies as are most required for some throats, but will allude to others hereafter under other heads. Among these we will merely mention at present *Calcarea carbonica, Graphites, Argentum, Cina,* and Digitalis, which are equally important for some of the complications of angina.—A. Gerald Hall, M.D., in *Hom. Examiner.*

